# Acknowledgement to Reviewers of *Nanomaterials* in 2014

**DOI:** 10.3390/nano5010061

**Published:** 2015-01-07

**Authors:** Nanomaterials Editorial Office

**Affiliations:** MDPI AG, Klybeckstrasse 64, CH-4057 Basel, Switzerland

The editors of *Nanomaterials* would like to express their sincere gratitude to the following reviewers for assessing manuscripts in 2014:

Adamson, Douglas H.
Addleman, Raymond Shane
Akbar, Sheikh
Aksimentiev, Aleksei
Antony, Joseph
Ao, Zhimin
Asadollahbaik, Asa
Bach, Udo
Beccai, Lucia
Bergamaschi, Enrico
Bethanis, Kostas
Bhatia, Suresh
Bing, Kong Ling
Bousset, Luc
Brehm, Moritz
Cai, Zhongyu
Carrey, Julian
Causin, Valerio
Cavaliere, Sara
Cesteros, Yolanda
Chau, Ying
Chen, Jun
Cheng, Xuanhong
Cheng, Zhen
Chiang, Anthony S. T.
Chiang, Ming-Hsi
Chiou, Chyow-San
Constantoudis, Vasilios
Craciun, Monica
Cui, Hui-Wang
Dan, Zhenhua
Davies, Gemma-Louise
Decuzzi, Paolo
Denizot, B.
Dini, Danilo
Elayaraja, M.
Erdem, Emre
Fahlman, Bradley D.
Fan, Wei
Fauzi, Iliana
Fechler, Nina
Fierro, Vanessa
Fröhlich, Eleonore
Fujita, Satoru
Fujita, Takeshi
Galloway, Tamara S.
Ghebreyessus, Kesete
Giardina, Paola
Gibson, Elizabeth A.
Gill, Richie H. S.
Gombau, L.
Grady, Brian
Gun’ko, Yurii
Hadjipanayis, Costas G.
Han, Byungchan
Harvey, Stephen C.
Hausera, Charlotte A. E.
Haverkamp, Richard G.
Ho, Wen-Jeng
Holden, Patricia
Hou, Tuo-Hung
Huang, Chih-Ching
Hurt, Robert H.
Iannazzo, Daniela
Jessel, Nadia
Jiang, Junhua
Jin, Dayong
Jobic, Stéphane
Johnson, David W.
Jovanović, Boris
Kaehr, Bryan
Kanakaraju, Subramaniam
Kanamura, Kiyoshi
Karthaus, Olaf
Kermanizadeh, Ali
Kessler, Vadim
Khan, Mujibur R.
Khodadoust, Amid P.
Kim, Jong-Duk
Kobayashi, Takeshi
Kontturi, Eero
Kostiainen, Mauri A.
Kubicki, Jim
Kuzuya, Akinori
Larsen, Kim Lambertsen
Larue, Camille
Lattuada, Marco
Laurent-Brocq, Mathilde
Lazo, Noel D.
Lee, Sang-Yup
Lei, Tingjun
Lin, Hong-Ping
Lindén, Mika
Linder, Markus B.
Liu, Chunli
Liu, Junwen
Liu, Xiaogang
Lützenkirchen-Hecht, Dirk
Male, David K.
Martinez-Manez, Ramon
Master, Emma R.
Meiners, Silke
Mimeire, Meire
Minus, Marilyn L.
Mitraki, Anna
Miyoshi, Hirokazu
Moddel, Garret
Ng, Kee Woei
Nie, X.
Niikura, Kenichi
Nishiyama, Norikazu
Ogami, Akira
Ouldridge, Thomas
Paavilainen, Sami
Pang, Xinchang
Parajuli, Durga
Park, Jeong-Sook
Patil, Avinash J.
Pericàs, Miquel A.
Piccaluga, Giorgio
Poland, Craig
Porter, Dale W.
Raghunathan, Vijay Krishna
Ravindra, Nuggehalli
Rumpf, K.
Sadhukha, Tanmoy
Schiraldi, David
Schladitz, Katja
Schreiber, Robert
Sevilla, Marta
Shah, Vishal
Shao, Yong
Slomberg, Danielle
Smirnova, E. A.
Song, Jenn-Ming
Steinbeck, Marla J.
Sugiyama, Hiroshi
Suh, Yung Doug
Sun, I.-W.
Tailhades, Philippe
Thiel, Werner R.
Tieu, Anh Kiet
Torrado, Juan J.
Tran, Phuoc X.
Tucek, Jiri
Valiullin, Rustem
Walker, Sharon L.
Wang, David
Wang, Erkang
Wang, Feihu
Wang, Xiao-Qian
Wang, Yan Mei
Wang, Ying-Jan
Whitea, Jason C.
Woo, Sungwook
Wu, Kevin
Xia, Tian
Xin, Xukai
Yallapu, Murali M.
Yamaguchi, Akira
Yamauchi, Yusuke
Yang, Mo
Yavuz, Cafer
Yigit, Mehmet V.
Yoo, Dongwon
Zampetti, E.
Zaraska, Leszek
Zhang, Hong
Zhang, Sanliang
Zhu, Yinghuai
Zinchenko, Anatoly

